# Crisdesalazine alleviates inflammation in an experimental autoimmune encephalomyelitis multiple sclerosis mouse model by regulating the immune system

**DOI:** 10.1186/s12868-024-00920-w

**Published:** 2025-01-03

**Authors:** Su-Min Park, Yong-Hun Oh, Ga-Hyun Lim, Ju-Hyun An, Jin-Hwan Lee, Byoung-Joo Gwag, So-Jung Won, Kyoung-Won Seo, Hwa-Young Youn

**Affiliations:** 1 Laboratory of Veterinary Internal Medicine, Department of Clinical Veterinary Science, College of Veterinary Medicine, Seoul National University, Seoul, 08826 Republic of Korea; 2 Department of Veterinary Emergency and Critical Care Medicine and Institute of Veterinary Science, College of Veterinary Medicine, Kangwon National University, Chuncheon-si, Republic of Korea; 3 GNT Pharma Co. Ltd., Yongin, Republic of Korea

**Keywords:** Anti-inflammation, Crisdesalazine, Experimental autoimmune encephalomyelitis, Microsomal prostaglandin E2 synthase-1, Macrophage

## Abstract

Microglia/macrophages participate in the development of and recovery from experimental autoimmune encephalomyelitis (EAE), and the macrophage M1 (pro-inflammatory)/M2 (anti-inflammatory) phase transition is involved in EAE disease progression. We evaluated the efficacy of crisdesalazine (a novel microsomal prostaglandin E2 synthase-1 inhibitor) in an EAE model, including its immune-regulating potency in lipopolysaccharide-stimulated macrophages, and its neuroprotective effects in a macrophage-neuronal co-culture system. Crisdesalazine significantly alleviated clinical symptoms, inhibited inflammatory cell infiltration and demyelination in the spinal cord, and altered the phase of microglial/macrophage and regulatory T cells. Crisdesalazine promoted the M1 to M2 phase transition in macrophages (immunomodulation) and reduced neuronal necrosis (neuroprotection) in vitro. This is the first study to directly demonstrate the therapeutic effects of a microsomal prostaglandin E2 synthase-1 inhibitor in an EAE model and its ability to alter macrophage polarization, suggesting that it may be a new therapeutic option for the treatment of patients affected by multiple sclerosis and other autoimmune diseases.

## Introduction

Multiple sclerosis (MS) is a neurodegenerative disease characterized by chronic inflammation of the brain and spinal cord [1]. Experimental autoimmune encephalomyelitis (EAE) is a mouse model of MS developed to investigate the clinical and immunological features of various autoimmune and neuroinflammatory diseases [2]. In EAE, activated T cells target the central nervous system (CNS) and trigger an inflammatory cascade, resulting in CNS infiltration and activation of immune cells [3]. This inflammatory process leads to the demyelination of nerve axons and the subsequent blockade of signal conduction at the site of demyelination [4]. Microglia/macrophages are closely involved in the pathogenesis of both MS and EAE [5, 6]. In the early phase of the disease, microglia/macrophages are typically in the M1 phase. M1 phase macrophages induce inflammation, axonal damage, and demyelination through the release of proinflammatory cytokines such as tumor necrosis factor-α (TNF-α) and interleukin-6 (IL-6). However, in the late phase, the microglia/macrophages change into the M2 phase; in this phase, they exert anti-inflammatory effects and promote tissue repair. Therefore, the microglial/macrophage M1/M2 phase transition plays an important role in the progression of EAE [7].

Crisdesalazine is a novel antioxidant drug candidate with beneficial effects in animal models of Alzheimer’s disease [8] and amyotrophic lateral sclerosis (ALS) [9]. In an ALS mouse model, crisdesalazine showed anti-inflammatory effects by inhibiting microsomal prostaglandin E2 synthase-1 (mPGES-1) [9]. mPGES-1 is the terminal enzyme in the biosynthesis of prostaglandin E2 (PGE2) and has recently been studied as a target for the selective inhibition of the PGE2 pathway [10]. PGE2 mediates inflammation, and several types of PGES enzymes are involved in the metabolic pathway responsible for the synthesis of PGE2 from prostaglandin H2. Among these enzymes, mPGES-1 is induced by inflammatory molecules such as lipopolysaccharide (LPS), interleukin-1b (IL-1b), and TNF-α [11]. The expression of mPGES-1 is linked to the synthesis of PGE2 mediated by cyclooxygenase 2 (COX-2), but other mPGES enzymes are also related to PGE2 production, regardless of inflammation status [12]. The role of mPGES-1 in inflammatory diseases, specifically in arthritis, has been studied using mPGES-1 knockout mice [13]. A previous study showed that mPGES-1 can be a target molecule to elicit anti-inflammatory effects in an EAE mouse model. mPGES-1 knockout mice show less severe symptoms and decreased production of inflammatory cytokines compared to wild type animals [14].

In the present study, we investigated the therapeutic efficacy and immunomodulatory capacity of crisdesalazine in a mouse EAE model. We also evaluated the immune-regulating ability of crisdesalazine in LPS-stimulated macrophages and its neuroprotective effect in a macrophage-neuronal co-culture system.

## Materials and methods

### EAE induction and therapy

This study was approved by the Institutional Animal Care and Use Committee (IACUC) of Seoul National University, Korea (SNU-220314-4). To induce EAE, we immunized 6-week-old female C57BL/6 mice with a myelin oligodendrocyte glycoprotein peptide (MOG_35-55_; Prospecbio, Israel) emulsified in complete Freund's adjuvant (CFA, Sigma-Aldrich, MO, USA) and administered pertussis toxin as described previously [2]. This protocol was carried out in accordance with established methods to ensure reproducibility of the EAE model. Crisdesalazine was supplied by GNT Pharma. A dimethylsulfoxide (DMSO) stock solution (500 mM) of the test compound was prepared and diluted in Dulbecco’s phosphate buffered saline (DPBS) at a concentration of 1 mM as the working solution for experiments. Crisdesalazine was intraperitoneally injected at a dose of 3.3 mg/kg q 24 h for 7 days starting at 9 days post EAE induction. In the control group, PBS was injected instead. The clinical signs pertaining to EAE were monitored daily and scored on a 0 to 5 scale as follows: grade 0, no obvious clinical symptoms; grade 0.5, partial tail paralysis; grade 1, tail paralysis or waddling gait; grade 1.5, partial tail paralysis and waddling gait; grade 2, tail paralysis and waddling gait; grade 2.5, partial limb paralysis; grade 3, paralysis of one limb; grade 3.5, paralysis of one limb and partial paralysis of another limb; grade 4, paralysis of two limbs; grade 4.5, moribund state; and grade 5, death. Symptoms were monitored until day 25, and euthanasia was performed. Before that, euthanasia was performed with a clinical score of 4.5 or higher. We used CO_2_ gas for euthanasia. CO_2_ was slowly introduced at a rate of 20% per minute, gradually increasing the concentration, and the animals were exposed to CO_2_ for 5 min. After confirming the loss of consciousness, cervical dislocation was performed to euthanize the animals.

### Histological analysis

Hematoxylin and eosin (H&E) and Luxol Fast Blue (LFB) staining were used to evaluate the degree of accumulation of inflammatory cells and that of demyelination. On day 25 post EAE induction, the mice were euthanized and the spleen and spinal cord were extracted. Paraffin-embedded spinal cords were sectioned at 5 μm thickness and stained with H&E and LFB. Histopathological examination was scored in a blinded manner as follows [15]: for inflammation: 0, no inflammatory cells; 1, a few scattered inflammatory cells; 2, organization of inflammatory infiltrates around blood vessels; and 3, extensive perivascular cuffing with extension into adjacent parenchyma, or parenchymal infiltration without obvious cuffing; for demyelination: 0, none; 1, rare foci; 2, a few areas of demyelination; and 3, large (confluent) areas of demyelination. Five serial sections of each spinal cord from eight mice per group were scored.

### Cell cultures

The human neuroblastoma cell line SH-SY5Y and the murine macrophage cell line RAW 264.7 were purchased from the Korean Cell Line Bank (KCLB, Korea). Both cell types were cultured in Dulbecco’s Modified Eagle Medium–high glucose (Welgene, Korea) supplemented with 100 units/mL penicillin G (Sigma-Aldrich), 100 μg/mL streptomycin (Sigma-Aldrich), and 10% fetal bovine serum (FBS; Gibco, USA), and then incubated at 37 °C in a humidified atmosphere containing 5% CO_2_.

RAW 264.7 macrophages were plated in 6-well plates (1.5 × 10^6^ cells/well) and treated with 200 ng/mL LPS for 6 h to induce the inflammatory phase. LPS-induced RAW 264.7 macrophages were treated with different concentrations of crisdesalazine (0.2, 1, and 5 μM) for 24 h.

### Cell viability assays

SH-SY5Y neurons and RAW 264.7 macrophages were cultured in 24-well plates at 2 × 10^5^ cells/well in a 500 μL volume and incubated for 24 h before addition of the reagents. Crisdesalazine was added to the wells at different final concentrations (0.2, 1, 5, 10, and 50 μM). The cytotoxic effect was calculated using the Cell Counting Kit-8 (CCK-8) assay (Dong-In biotech, Seoul, Korea).

### Co-culture with RAW 264.7 and SH-SY5Y

RAW 264.7 macrophages were cultured in culture inserts and treated with 200 ng/mL LPS for 6 h. The inserts were then transferred to 6-well plates containing SH-SY5Y neuronal cultures. The SH-SY5Y neurons were treated with different concentrations of crisdesalazine (0.2, 1, and 5 μM) and co-cultured with the RAW 264.7 cells for 24 h. In this co-culture method, macrophages can interact with neuronal cells through a semipermeable membrane, and there is no direct contact between the two cell cultures.

### RNA extraction, cDNA synthesis and real-time PCR

Total RNA was extracted from cells or tissues using the Easy-Blue RNA extraction kit (iNtRON Biotechnology, Korea) according to the manufacturer’s instructions. cDNA was synthesized using the Cell Script All-in-One 5X First Strand cDNA Synthesis Master Mix (Cell Safe, Korea). RNA expression was analyzed using 400 nM of forward and reverse primers (Bionics, Korea) and AMPIGENE qPCR Green Mix Hi-ROX with SYBR Green dye (Enzo Life Sciences, Farmingdale, NY, USA) in an Applied Biosystems™ QuantStudio 5 qPCR System (Thermofisher, USA). The expression level of each gene was normalized to glyceraldehyde 3-phosphate dehydrogenase (GAPDH) and compared with the expression levels recorded in the naïve or control group. The sequences for the primers used in the quantification are shown in Table 1.

**Table 1:** Sequences of PCR primers in this study

Species	Gene	Sequence
Mouse	iNOS	F: CCT CCT CCA CCC TAC CAA GT
		R: CAC CCA AAG TGC TTC AGT CA
	IL-6	F: AGT TGC CTT CTT GGG ACT GA
		R: TCC ACG ATT TCC CAG AGA AC
	IL-1β	F: TGG ACC TTC CAG GAT GAG GAC A
		R: GTT CAT CTC GGA GCC TGT AGT G
	CD206	F: AAC GGA ATG ATT GTG TAG TTC TAG C
		R: TAC AGG ATC AAT AAT TTT TGG CAT T
	Foxp3	F: TTG GCC AGC GCC ATC TT
		R: TGC CTC CTC CAG AGA GAA GTG
	TNF-α	F: CCC TCA CAC TCA GAT CAT CTT CT
		R: GCT ACG ACG TGG GCT ACA G
	IFN-γ	F: CTC TTC TTG GAT ATC TGG AGG AAC T
		R: GCT GTT GCT GAA GAA GGT AGT AAT C
	GAPDH	F: AGT ATG TCG TGG AGT CTA CTG GTG T
		R: AGT GAG TTG TCA TAT TTC TCG TGG T
Human	IL-6	F: CCC TGA CCC AAC CAC AAA TG
		R: CTA CAT TTG CCG AAG AGC CC
	IL-1β	F: GCT GGA ATT TGA GTC TGC CC
		R: TAT ATC CTG GCC GCC TTT GG
	TNF-α	F: CGC TCC CCA AGA AGA CAG G
		R: GGG GCC GAT CAC TCC AAA G
	GAPDH	F: AGG TCG GAG TCA ACG GAT TT
		R: TGA CGG TGC CAT GGA ATT TG

### Immunofluorescence analysis

For immunofluorescence (IF) staining, the cells were fixed with 4% paraformaldehyde and blocked with a buffer containing 5% bovine serum albumin (BSA; Sigma-Aldrich) and 0.1% Triton X-100 (Sigma-Aldrich) for 30 min. The cells were then incubated at 4 °C for 1 h with an allophycocyanin (APC)-conjugated antibody against CD11c (1:100; Santa Cruz Biotechnology, CA, USA) and a fluorescein isothiocyanate (FITC)-conjugated antibody against CD206 (1:100; Santa Cruz Biotechnology). After three washes, the cells were mounted with Vectashield mounting medium containing 4’,6-diamidino-2-phenylindole (DAPI; Vector Laboratories, Burlingame, CA, USA). The cells were observed under an EVOS FL microscope (Life Technologies, Darmstadt, Germany).

### Protein extraction and Western blotting

Proteins were extracted using the Pro-Prep protein extraction solution (Intron Biotechnology, Korea). Protein concentration in the samples was determined using a DC Protein Assay Kit (Bio-Rad, Hercules, CA, USA). For Western blot assays, 25 μg of protein was subjected to sodium dodecyl sulfate–polyacrylamide gel electrophoresis (SDS-PAGE). The separated proteins were transferred to polyvinylidene difluoride membranes (EMD Millipore, Billerica, MA, USA). The membranes were blocked with 5% BSA in Tris-buffered saline and incubated at 4 °C overnight with primary antibodies against NF-kB (1:1000; Cell Signaling Technology, USA) and β-actin (1:1000, Santa Cruz Biotechnology). The membranes were then washed several times and further incubated with the appropriate secondary antibody in 5% non-fat dry milk for an hour. Immunoreactive bands were detected using an enhanced chemiluminescence detection kit (Advansta, Menlo Park, CA, USA) and normalized to the band intensity observed for β-actin.

### Annexin V

SH-S5Y5 cells co-cultured with RAW 264.7 were stained with annexin V-fluorescein isothiocyanate/propidium iodide (PI) according to the manufacturer’s instructions (BD Biosciences, San Jose, CA, USA), and subsequently analyzed using a fluorescence-activated cell sorting instrument (FACS Aria II; BD Biosciences, San Jose, CA, USA). The stained cells were categorized as follows: PI- and annexin V-negative (lower left quadrant), normal; PI-negative and annexin V-positive (lower right quadrant), early apoptotic; PI- and annexin V-positive (upper right quadrant), late apoptotic; and PI-positive and annexin V-negative (upper left quadrant), necrotic.

### Splenocyte isolation and activation

The experimental animals were sacrificed on day 25 post EAE induction. Splenocytes were isolated using a 100 μm cell strainer (SPL, Korea). RBCs were eliminated using a RBC lysis buffer, and the splenocytes were cultured in Roswell Park Memorial Institute-1640 medium (Welgene, Korea) supplemented with 10% FBS (Gibco), 100 units/mL penicillin G (Sigma-Aldrich), and 100 μg/mL streptomycin (Sigma-Aldrich). To evaluate antigen-specific reactions, splenocytes were activated ex vivo using 10 ug/mL MOG_35-55_ (Prospecbio, Israel) for 48 h.

### Flow cytometric analysis of regulatory T cells in activated splenocyte preparations

To evaluate the effect of crisdesalazine on regulatory T cells (Tregs), mouse splenocyte preparations were stained with a Treg Detection Kit (CD4/CD25/FoxP3) (Miltenyi Biotech, Germany) according to the manufacturer’s instructions, and the Treg population was evaluated using flow cytometry. Briefly, activated splenocytes from the mice (1 × 10^6^) were washed and stained with specific monoclonal antibodies as follows: the cells were incubated with a FITC-conjugated anti-CD4 and a PE-conjugated anti-CD25 antibody at 4 °C for 30 min. The cells were then washed, fixed, and permeabilized for the intracellular staining of Foxp3 using an APC-conjugated anti-Foxp3 antibody. The cells were subjected to flow cytometry and the data were analyzed using the FlowJo™ software (version 10.8.1; BD Biosciences, San Jose, CA, USA). First, CD4 + lymphocytes were gated, and among the gated CD4 + cells, CD25 + FoxP3 + cells were designated as Tregs.

### Cytokine assay

Cytokine production from activated splenocytes was measured in the cell culture medium. The IL-6 concentration was measured using a commercial ELISA kit (mouse IL-6 kit; Invitrogen, MA, USA) according to the manufacturer’s instructions.

### Statistical analyses

Data are shown as the mean ± standard deviation. Mean values from different groups were compared using the Mann–Whitney t-test and one-way analysis of variance. All statistical comparisons were performed using GraphPad Prism (version 7.01; GraphPad Software, La Jolla, CA, USA). Statistical significance was set at P < 0.05.

## Results

### Effects of crisdesalazine on EAE clinical signs and histological changes in the spinal cord

**Fig. 1:**
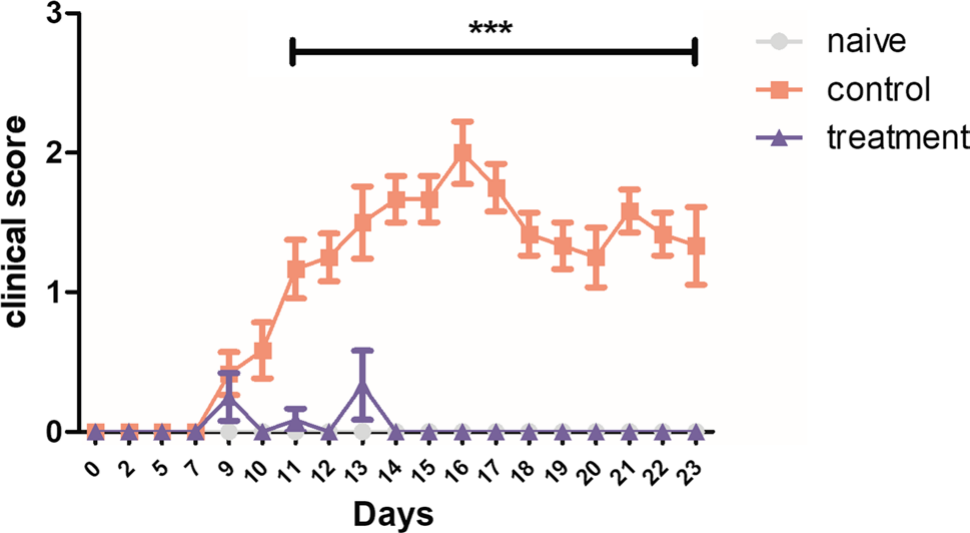
Clinical scores pertaining to the EAE mouse model. The clinical score of the control group increased significantly from day 11 to day 23 post-induction. The clinical signs were alleviated effectively in the crisdesalazine treatment group. ***P < 0.001

Clinical symptoms were evaluated daily; the onset of EAE-related neurological signs was observed on day 9, and symptom severity reached its peak on day 16 (Fig. 1). The day of onset was similar in the EAE and the EAE + crisdesalazine groups. However, crisdesalazine treatment significantly reduced symptom severity after onset (Fig. 1). No drug-related adverse effects were observed in the treated group.

**Fig. 2:**
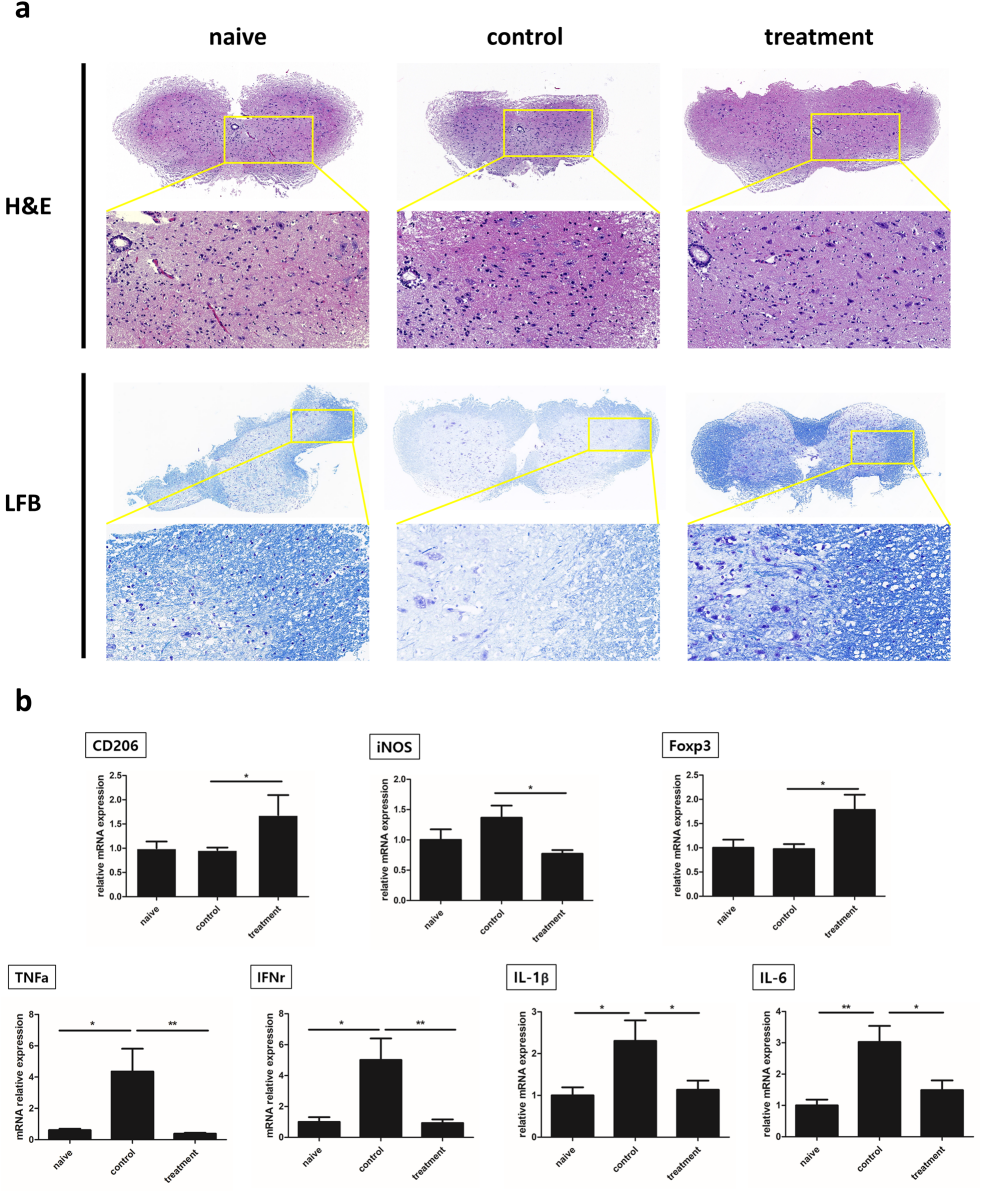
Histopathological evaluation and changes in cytokine expression in the spinal cord of EAE mice. **a** Hematoxylin and eosin (H&E) staining (upper & middle lane) shows that cell infiltration was significantly increased in the control group. Disseminated inflammatory cells were observed in the spinal cord of the control group. Infiltration of inflammatory cells was decreased in the treatment group. Luxol fast blue (LFB) staining (lower lane) shows demyelination of the spinal cord in the control group; the demyelination area was lower in the treatment group. **b** Expression of cytokines in the spinal cord. The levels of CD206 and Foxp3 were significantly higher in the treatment group. In the control group, the levels of the inflammatory cytokines TNF-α, IFN-γ, IL-1β and IL-6 were significantly increased. In contrast, inflammatory cytokines in the spinal cord were decreased in the treatment group. Results are shown as means ± standard deviation. *P < 0.05, ** P < 0.01, ***P < 0.001; ns, not significant

On day 25 post EAE induction, the mice were euthanized, and the spinal cord was isolated from each animal. To evaluate inflammatory cell infiltration, spinal cord sections were stained with H&E. Mononuclear cell infiltration in the spinal cord was elevated in the EAE group compared to the naïve group (Fig. 2A) and significantly reduced in the EAE + crisdesalazine group (Fig. 2A). Moreover, demyelination areas were significantly increased in the EAE group, as indicated by the LFB staining in the spinal cord sections. Compared to the EAE group, the EAE + crisdesalazine group exhibited attenuated demyelination (Fig. 2A).

The expression of CD 206, a M2 anti-inflammatory phase marker, was significantly increased, and that of inducible nitric oxide synthase (iNOS), a M1 pro-inflammatory phase marker, was significantly decreased in the EAE + crisdesalazine group (Fig. 2B). Foxp3, a Treg cell marker, was highly expressed in the crisdesalazine-treated group (Fig. 2B). The expression of TNF-α, interferon-γ (IFN-γ), IL-1β, and IL-6 (potent macrophage/lymphocyte-related pro-inflammatory cytokines) was also decreased in animals treated with crisdesalazine (Fig. 2B).

### Crisdesalazine alters T cell populations and cytokine expression in the spleen

**Fig. 3:**
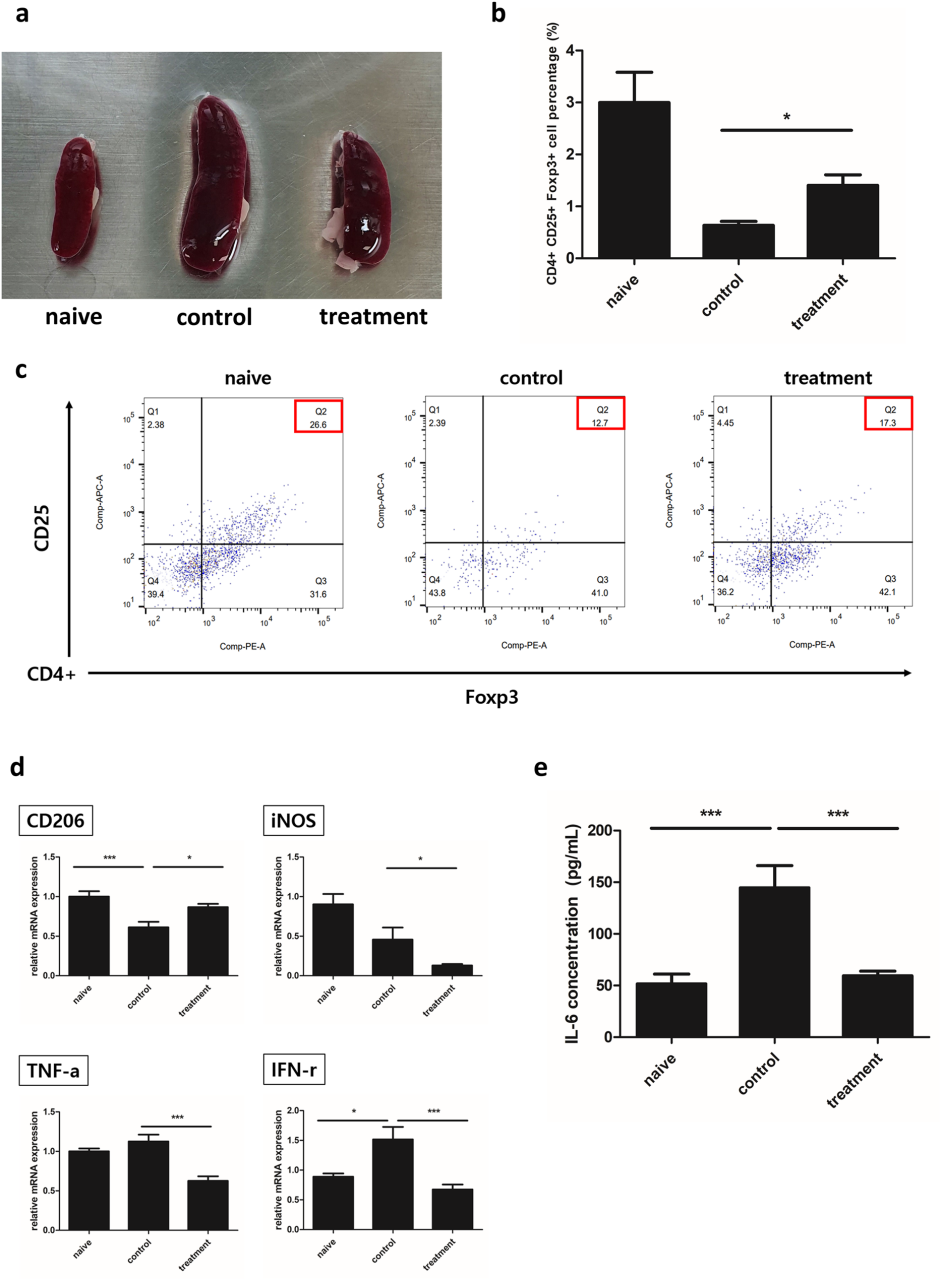
Analysis of the regulatory T cell (Treg) population, RNA expression, and cytokines in the spleen tissue of EAE mice.** a** Spleens were enlarged in the control EAE group, whereas the size of the spleen in the treatment group was similar to that in the naïve group. **b**, **c** The proportion of CD4 + CD25 + Foxp3 + Treg cells was lower in the control group compared to the naïve group; the proportion of this population was higher in the treatment group compared to that in the control group. **d** The expression of CD206 was decreased in the control EAE group, but significantly increased in the treatment group compared to that in the control group. The expression of iNOS was significantly decreased in the treatment compared to that in the control group. The expression of the inflammatory cytokines tumor necrosis factor alpha (TNF-α) and interferon gamma (IFN-γ) was lower in the treatment group compared to that in the control group. **e** Interleukin 6 (IL-6) concentration was measured in activated splenocytes and found to be lower in the treatment group compared to that in the control group. Results are shown as means ± standard deviation. *P < 0.05, ***P < 0.001

On day 25 post EAE induction, spleens were isolated from the animals and dissected; splenocytes were isolated after erythrocyte lysis. The EAE group exhibited significant spleen enlargement compared to the naïve group, whereas the EAE + crisdesalazine group exhibited a reduction in spleen size (Fig. 3A).

To evaluate the effect of crisdesalazine on Treg cells, CD4 + CD25 + Foxp3 + T cells (Treg subsets) were analyzed in splenocytes activated by MOG_35-55_ treatment. The percentage of Treg cells in the EAE group was significantly lower than that in the naïve group. In contrast, the Treg cell population was significantly increased in the crisdesalazine-treated group (Fig. 3B, C).

We also analyzed cytokine expression in the splenocyte preparations. The expression of CD206 was significantly increased in the EAE + crisdesalazine group compared to that in the EAE group (Fig. 3D). The expression of TNF-α and IFN-γ (both related to inflammation and immune response) was significantly decreased in the EAE + crisdesalazine group (Fig. 3D).

To evaluate changes in inflammatory cytokine secretion after crisdesalazine treatment, we measured IL-6 levels in the growth media of MOG-activated splenocytes. The IL-6 levels were higher in the EAE group (51.66 ± 9.454 pg/mL) than in the naïve group (144.6 ± 21.55 pg/mL). On the other hand, in the crisdesalazine-treated group, the IL-6 levels were significantly reduced (59.35 ± 4.524 pg/mL) and similar to those in the naïve group (Fig. 3E).

### *Changes in macrophage polarization with crisdesalazine treatment *in vitro

**Fig. 4:**
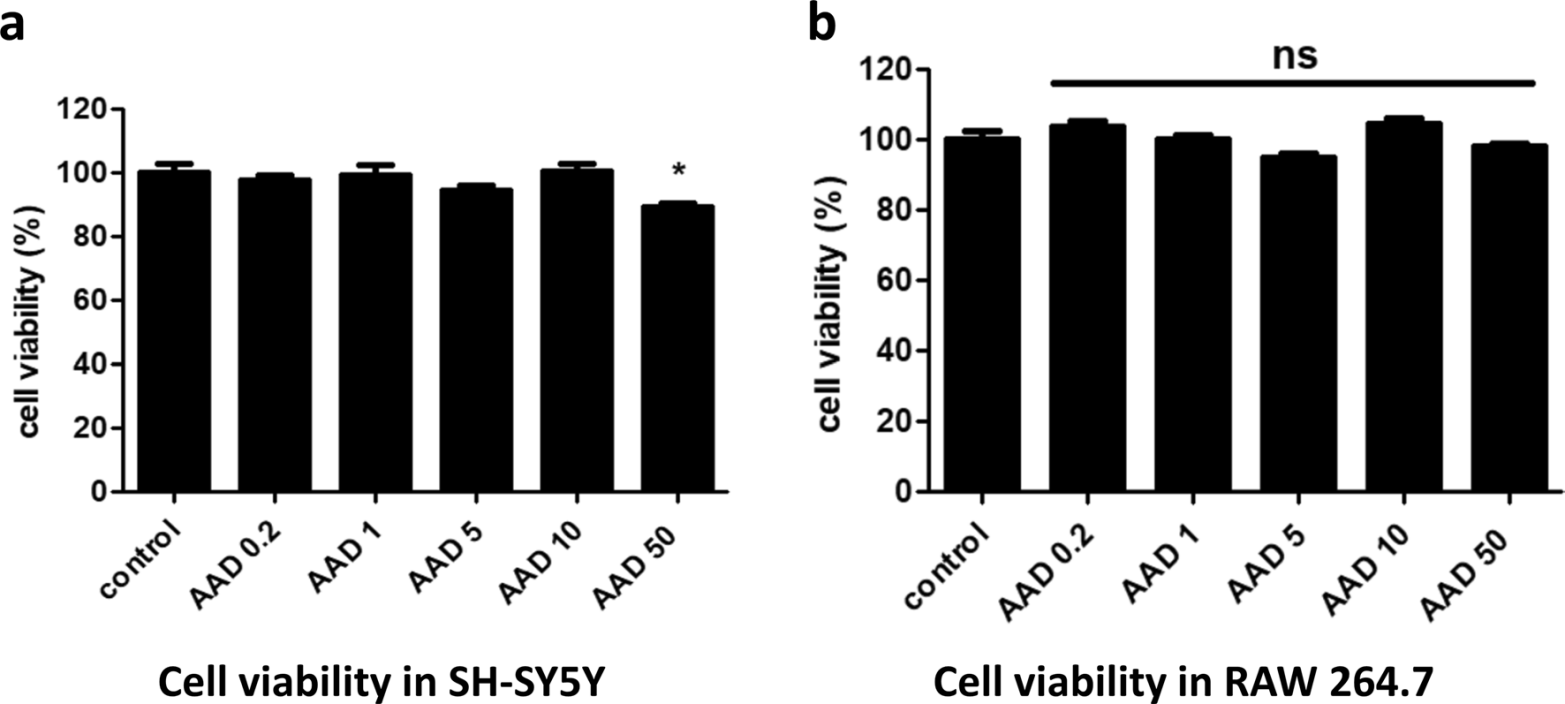
Cell viability assessment using the CCK-8 assay in SH-SY5Y neuronal cells and RAW 264.7 macrophage cells. **a**, **b** The viability of either cell type was not affected at a crisdesalazine concentration of < 10 μΜ, but that of SH-SY5Y cells was decreased at a concentration of 50 μΜ. Results are shown as means ± standard deviation. *P < 0.05; ns, not significant

The cytotoxicity of crisdesalazine was evaluated by assessing the cell viability of RAW264.7 and SH-SY5Y cells treated with the drug. The viability of the SH-SY5Y cells was decreased upon treatment with 50 μM crisdesalazine (Fig. 4A); however, the viability of the RAW 264.7 cells was not affected by crisdesalazine at concentrations < 50 μM (Fig. 4B). Therefore, we decided to use crisdesalazine at concentrations below 50 μM in the in vitro study.

**Fig. 5:**
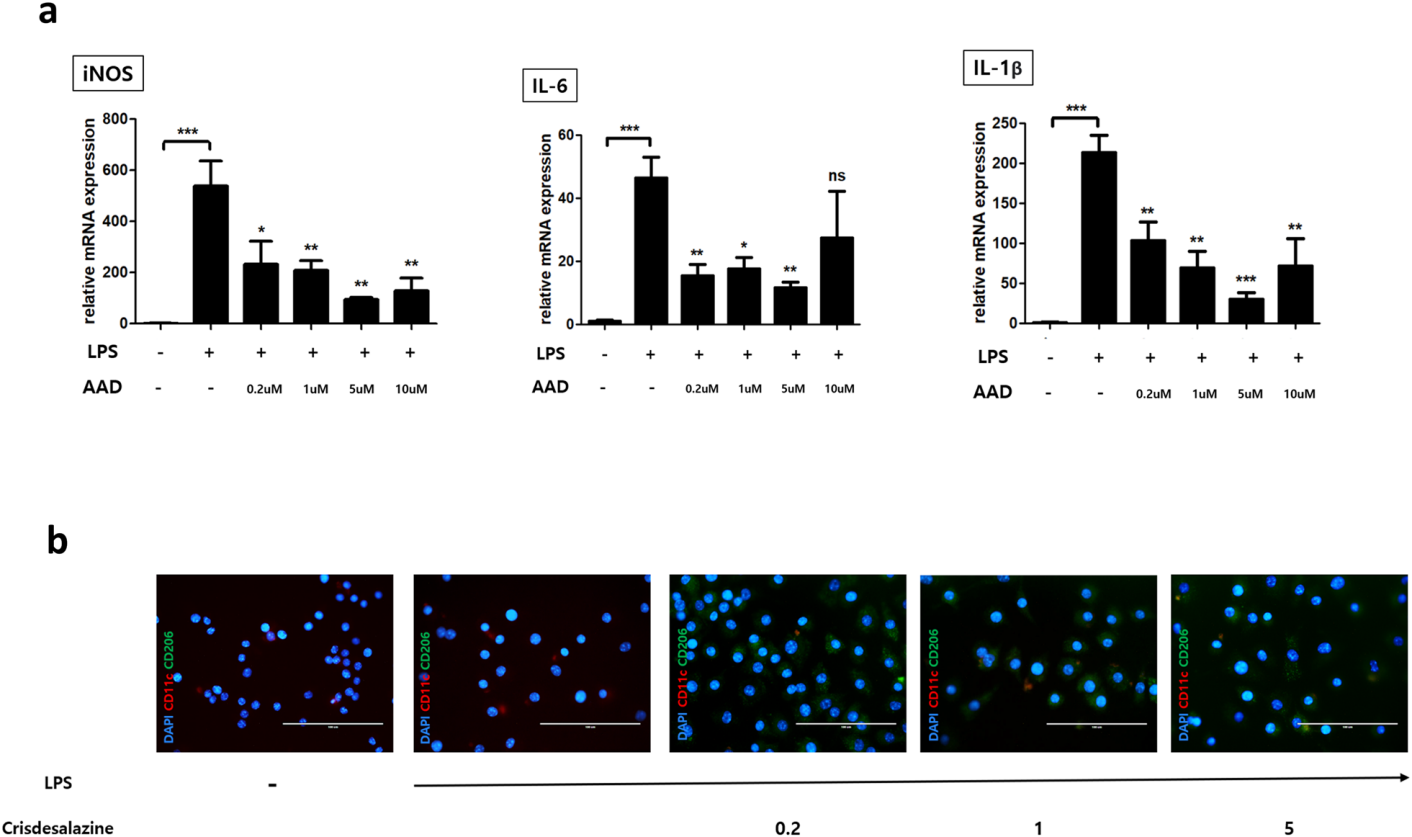
Changes in macrophage polarization from the proinflammatory M1 to the anti-inflammatory M2 phases due to crisdesalazine treatment. **a** mRNA expression levels of iNOS, a M1 macrophage marker. Proinflammatory M1 phase was induced when macrophages were stimulated with lipopolysaccharide (LPS) and was reversed when they were treated with crisdesalazine. There was a significant increase in interleukin 6 (IL-6) and interleukin 1β (IL-1β) expression after stimulation with LPS. When LPS-stimulated macrophages were treated with crisdesalazine, the expression levels of both cytokines were reduced, with the exception of IL-6 at 10 μΜ crisdesalazine. **b** Detection of CD11c + (M1 marker, red) cells and CD206 + (M2 marker, red) cells using an immunofluorescence assay. In the crisdesalazine-treated group, the proportion of CD206 + cells increased compared to the untreated group. Results are shown as means ± standard deviation. *P < 0.05, ** P < 0.01, ***P < 0.001; ns, not significant

To evaluate the effect of crisdesalazine in macrophages, RAW 264.7 cells were stimulated with LPS to induce the M1 phase. After stimulation with LPS, the expression of the M1 pro-inflammatory marker iNOS and the expression of the pro-inflammatory cytokines IL-1β and IL-6 were significantly increased in these cells (Fig. 5A). In contrast, in groups treated with crisdesalazine (0.2, 1, and 5 μM), the expression of iNOS, IL-1β, and IL-6 was significantly decreased compared to that in the untreated group (Fig. 5A). However, there was no significant difference in IL-6 expression in the group treated with 10 μM crisdesalazine compared to the untreated group (Fig. 5A). Therefore, we decided to exclude this concentration (10 μM) in the subsequent experiments.

We evaluated changes in macrophage polarization induced by crisdesalazine treatment based on the immunofluorescent staining of CD11c (red stain) and CD206 (green stain) (markers of M1 and M2 phases, respectively). CD11c expression increased in the LPS-stimulated group. Crisdesalazine-treated groups showed significantly increased CD206 staining compared to the untreated groups (Fig. 5B).

### *Crisdesalazine reduces neuro-inflammation in an *in vitro* co-culture system*

**Fig. 6:**
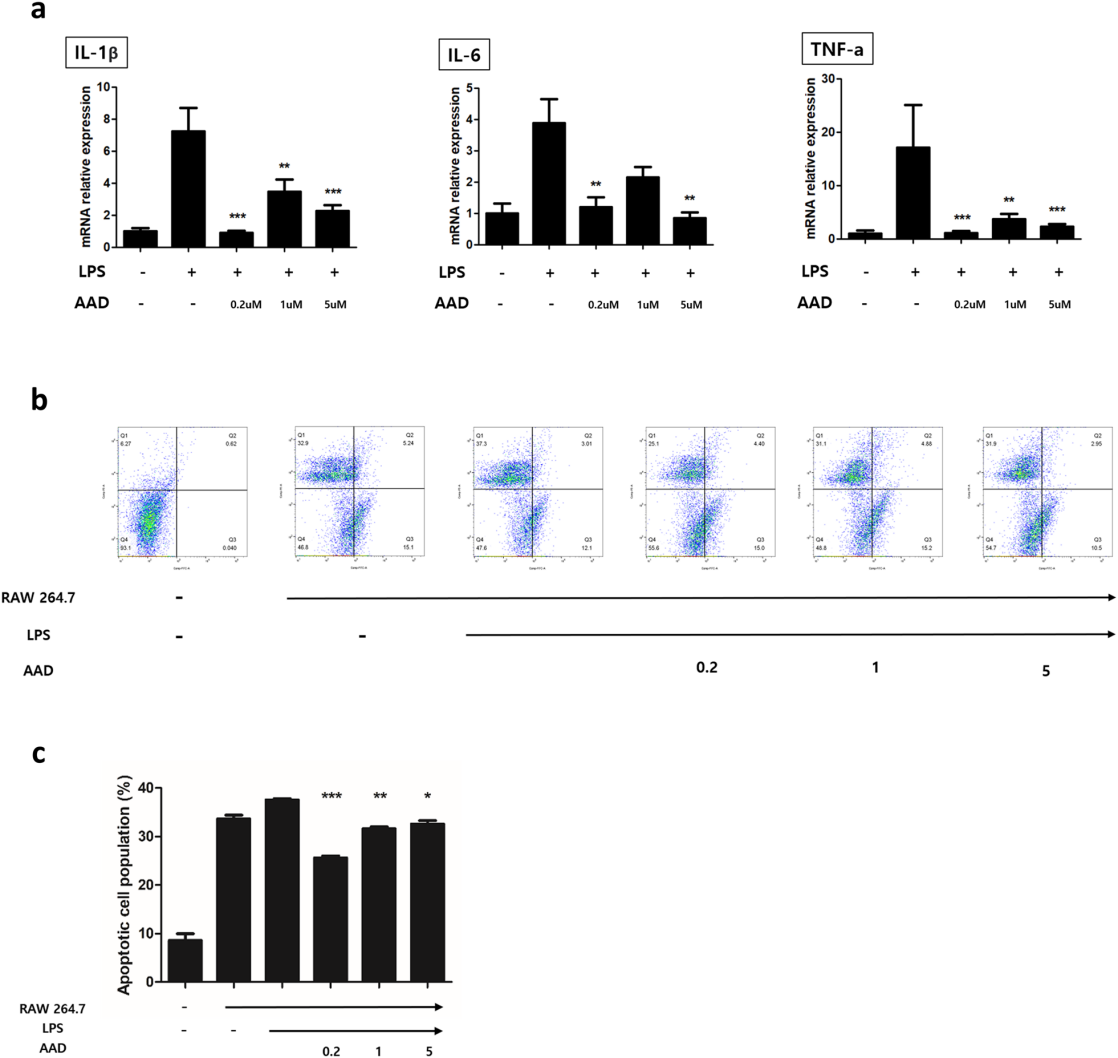
Crisdesalazine treatment reduces neuroinflammation and neuronal necrosis in a macrophage-neuronal co-culture system.** a** mRNA expression levels of pro-inflammatory factors in SH-SY5Y neuron cells which were co-cultured with lipopolysaccharide (LPS)-stimulated macrophages. Compared to the untreated group, the expression of interleukin 1β (IL-1β), interleukin 6 (IL-6), and tumor necrosis factor alpha (TNF-α) were significantly decreased in the crisdesalazine-treated group. **b**, **c** Co-culture groups that were not treated with crisdesalazine exhibited a significant increase in neuronal necrosis compared to the naïve group. The crisdesalazine-treated group showed reduced neuronal necrosis compared to the group that was not treated with crisdesalazine. Results are shown as means ± standard deviation. *P < 0.05, ** P < 0.01, ***P < 0.001

To investigate whether crisdesalazine protects neuronal cells from inflammation induced by macrophages, a co-culture system with SH-SY5Y and RAW 264.7 cells was used. When SH-SY5Y cells were co-cultured with LPS-treated RAW 264.7 cells, the expression of IL-1β, IL-6, and TNF-α was significantly increased compared to that in the control group (which was co-cultured with non-stimulated macrophages; Fig. 6A). In contrast, in the crisdesalazine-treated groups, the expression of IL-1β, IL-6, and TNF-α was significantly decreased (Fig. 6A). In particular, the expression level of inflammatory cytokines was markedly decreased upon treatment with 0.2 and 5 μM crisdesalazine.

Annexin V-FITC analysis was performed to evaluate necrosis of SH-SY5Y cells under inflammatory conditions. Compared with naïve SH-SY5Y cells without co-culture, SH-SY5Y cells that were co-cultured with macrophages exhibited significantly higher levels of necrosis (Fig. 6B). There was no significant difference in the levels of necrosis among the crisdesalazine-treated groups regardless on whether the macrophages had been treated with LPS or not. However, necrosis was significantly reduced in crisdesalazine-treated SH-SY5Y groups. Of note, necrotic cell populations were markedly reduced in the 0.2 μM crisdesalazine treatment group (Fig. 6C).

## Discussion

Crisdesalazine prevents oxidative stress by acting as a spin-trapping agent. It prevents inflammation by inhibiting mPGES-1, an inducible enzyme essential for PGE2 production [9]. Several studies have shown that mPGES-1 has the potential to modulate immune cells [14, 16, 17]. However, a few studies have reported that mPGES-1 inhibitors are not efficacious in autoimmune diseases. Thus, we investigated the clinical effect of crisdesalazine in an EAE mouse model and in an in vitro macrophage model.

In the present study, we confirmed that clinical symptoms were alleviated when crisdesalazine was administered to EAE mice, and that both the infiltration of inflammatory cells and the demyelination were reduced according to the histological evaluation of the spinal cord. The expression of inflammatory cytokines in spinal cord tissue was also reduced in the EAE + crisdesalazine group. Moreover, the expression and secretion of inflammatory cytokines in the spleen, a representative peripheral lymphatic organ, were significantly reduced in the EAE + crisdesalazine group. These pieces of evidence imply that crisdesalazine has a therapeutic effect in the EAE model. Interestingly, crisdesalazine treatment led to a significant decrease in the expression of iNOS, a marker for the M1 phenotype, and a significant enhancement in the expression of CD206, a marker for the M2 phenotype, compared to the EAE group. In addition, the crisdesalazine-treated groups exhibited an increase in the expression of Foxp3 (a marker for Treg cells) in the spinal cord and spleen. PGE2 promotes the differentiation and expansion of inflammatory Th1 and Th17 cells and facilitates T cell-mediated immune inflammation, and thus EAE progression [18, 19]. Whether PGE2 activates or inhibits Tregs is still controversial, but studies have shown that PGE2 inhibits Treg differentiation [20–22] and that PGE2 inhibition using non-steroidal anti-inflammatory drugs (NSAIDs) activates Treg cells [23, 24]. Considering these findings together with the results of the current study, it may be concluded that crisdesalazine (acting as a mPGES-1 inhibitor) shows therapeutic effects in EAE through its immunomodulatory activity based on the inhibition of PGE2 production, restriction of Th1 and Th17 cells, and activation of Treg cells.

Additionally, no adverse gastrointestinal effects were observed. The standard method for suppressing the production of PGE2 is inhibiting COX, the primary enzyme responsible for PGE2 production. However, this inhibition leads to the non-selective suppression of other prostaglandins as well; therefore, COX inhibitors cause adverse events such as gastrointestinal damage, decreased renal function, and cardiovascular problems. For this reason, several studies have targeted mPGES-1 in an attempt to reduce side effects [14, 25, 26]. In this study, crisdesalazine exhibited therapeutic effects with negligible adverse events.

The immunomodulatory effects of crisdesalazine were evaluated in RAW 264.7, a murine macrophage cell line. Crisdesalazine promoted M1 to M2 phase transition and suppressed the expression of inflammatory cytokines in LPS-stimulated macrophages. In the macrophage-neural cell co-culture system, both necrosis and the expression of inflammatory cytokines in the neuronal cells were reduced after crisdesalazine treatment in a dose-dependent manner. Interestingly, neural cell necrosis was induced by co-culture with macrophages that had not been stimulated with LPS. According to Pandur et al., the viability of SH-SY5Y cells under LPS treatment was further reduced in the group co-cultured with the murine microglial cell line BV2 compared to that in the monoculture group. [27]. It could be inferred that co-culture with microglia/macrophages is associated with neuronal survival and that the infiltration of these inflammatory cells alone may cause neuronal inflammation and necrosis. Thus, crisdesalazine potentially exhibits neuroprotective activity by modulating the macrophage phenotype and inhibiting the infiltration of inflammatory cells.

Previous studies have shown that macrophages/microglia play an important role in the pathogenesis and progression of MS and EAE [6, 28, 29]. During the induction phase of EAE, microglia and the infiltrated macrophages in the CNS are activated, becoming a major source of various cytokines, chemokines, and ROS; directly damaging CNS tissue, and amplifying inflammation [30–32]. M2 macrophages secrete anti-inflammatory cytokines to reduce inflammatory infiltration and promote the differentiation of Th2 and Treg cells, which play an important role in controlling inflammation [32]. The transition between the M1 and M2 phenotypes of macrophages/microglia is reversible, and the precise ratio between both phenotypes may promote or inhibit EAE progression [33]. Therefore, the control of inflammatory cells has become the focus of EAE treatment, and we show here the possibility of applying crisdesalazine as a novel immunomodulatory drug.

mPGES-1 is a key factor in macrophage regulation. The upregulation of mPGES-1 in macrophages results in the production of PGE2 during inflammatory activation by pro-inflammatory stimuli such as LPS, IL-1β, or TNFα, and these effects are opposed by the inhibitory actions of glucocorticoids [34]. Mosca et al. reported that in humans, activated mononuclear phagocytes in the M1 phase (in the peripheral blood) express mPGES-1, but those in the M2 phase do not [35]. Furthermore, PGE2 initiates a positive feedback loop of COX-2 and mPGES-1 expression in macrophages [36]. In LPS-stimulated macrophages, COX-2 induces PGE2 production, whereas a subsequent increase in PGE2 levels is essentially mPGES-1-driven [37]. Thus, it is important to downregulate mPGES-1 to disrupt the inflammatory feedback mechanism. In the present study, we found that crisdesalazine inhibited mPGES-1 in macrophages and disrupted the positive feedback loop of PGE2 and mPGES-1; this promoted the transition of macrophages into the M2 (anti-inflammatory) phase.

In this study, the immunomodulatory activity of crisdesalazine was investigated only in macrophages, while such effects in responder cells such as T cells was not elucidated at the molecular level. Therefore, additional research is needed on the mechanism underlying this immunomodulation. However, the increase in Treg cells evidenced by the analysis of the spleen in the EAE model suggests that the anti-inflammatory pathway may be boosted by the inhibition of mPGES-1 in T cells.

To the best of our knowledge, this is the first study to directly demonstrate the therapeutic effects of a mPGES-1 inhibitor in an EAE mouse model, and its effect on macrophage polarization. Crisdesalazine ameliorates neuronal inflammation by regulating immune cells in the anti-inflammatory phase. This result suggests that crisdesalazine may be a new therapeutic option for MS and other autoimmune diseases.

## Data Availability

All data generated or analyzed during this study are included in this published article.

## Abbreviations

EAE: Experimental autoimmune encephalomyelitis
mPGES-1: Microsomal prostaglandin E2 synthase-1
LPS: Lipopolysaccharide
Tregs: Regulatory T cells
MS: Multiple sclerosis
CNS: Central nervous system
IL: Interleukin
TNF-α: Tumor necrosis factor-α
ALS: Amyotrophic lateral sclerosis
PGE2: Prostaglandin E2
COX-2: Cyclooxygenase 2
MOG_35-55_: Oligodendrocyte glycoprotein peptide myelin oligodendrocyte glycoprotein_35-55_
CFA: Complete Freund’s adjuvant
DMSO: Dimethylsulfoxide
DPBS: Dulbecco’s phosphate buffered saline
H&E: Hematoxylin and eosin
LFB: Luxol Fast Blue
FBS: Fetal bovine serum
CCK-8: Cell Counting Kit-8
GAPDH: Glyceraldehyde 3-phosphate dehydrogenase
IF: Immunofluorescence
BSA: Bovine serum albumin
DAPI: 4’,6-Diamidino-2-phenylindole
APC: Allophycocyanin
FITC: Fluorescein isothiocyanate
SDS-PAGE: Sodium dodecyl sulfate-polyacryalmide gel electrophoresis
PI: Propidium iodide
FACS: Fluorescence-activated cell sorting
iNOS: Inducible nitric oxide synthase
NSAIDs: Non-steroidal anti-inflammatory drugs
